# Effect of arsenic stress on the intestinal structural integrity and intestinal flora abundance of *Cyprinus carpio*

**DOI:** 10.3389/fmicb.2023.1179397

**Published:** 2023-04-24

**Authors:** Xiaodan Shi, Wei Xu, Xinghua Che, Jiawen Cui, Xinchi Shang, Xiaohua Teng, Zhiying Jia

**Affiliations:** ^1^Heilongjiang River Fisheries Research Institute, Chinese Academy of Fishery Sciences, Harbin, China; ^2^Key Laboratory of Freshwater Aquatic Biotechnology and Breeding, Ministry of Agriculture and Rural Affairs, Heilongjiang Fisheries Research Institute, Chinese Academy of Fishery Sciences, Harbin, China; ^3^College of Animal Science and Technology, Northeast Agricultural University, Harbin, China

**Keywords:** arsenic, accumulation, intestinal microbiota, LPS, tight junction

## Abstract

Aquatic organisms such as fish can accumulate high concentrations of arsenic (As), which has toxic effects on fish. However, whether the intestinal flora are involved in As damage to fish intestinal tissues and the underlying process are unclear. Common carp (Cyprinus *carpio*) were exposed to As (2.83 mg/L) in water for 30 days, and blood, muscle, intestine, and intestine samples were collected. Intestinal pathological sections were observed, and the lipopolysaccharide (LPS) levels in serum and the levels of As accumulation and tight junction-related factors in intestinal tissues were measured. The gut microbiota was analysed by 16S rRNA sequencing. The results showed that As treatment decreased the abundance of microbiota, increased the number of harmful bacteria, and decreased the number of beneficial bacteria in the intestine. In our experiment, the top 30 harmful and beneficial bacteria with the highest relative abundance were identified. Among the top 30 harmful and beneficial bacteria, As treatment resulted in a significant (*P* < 0.05) increase in harmful bacteria (such as *Fusobacteriota, Bacteroidota* (LPS-producing bacteria), *Verrucomicrobiota, Bacteroides, Aeromonas*, and *Stenotrophomonas*) and a significant (*P* < 0.05) decrease in beneficial bacteria (such as *Actinobacteriota, Planctomycetota, Firmicutes, Reyranella, Akkermansia*, and *Pseudorhodobacter*), which further demonstrated that As affects the abundance of intestinal flora. In addition, As exposure increased the LPS level in serum and the abundance of *Bacteroidota* (LPS-producing bacteria) in the intestine. *Bacteroidota* exhibits the six highest relative abundance at the phylum level, which indicates that LPS produced by *Bacteroidota* can increase the LPS level in serum. Additionally, the protein and gene levels of the tight junction markers ZO-1 and *occludin* in the intestine were reduced by As treatment, which further indicated that As exposure impaired the structural integrity of the intestine. In conclusion, the results obtained in our study indicate that the intestinal flora, LPS, and tight junctions participate in the impairment of the structural integrity of the common carp intestine resulting from As exposure.

## 1. Introduction

Heavy metals in the aquatic environment can cause damage to fish. Hexavalent chromium exposure causes chromium bioaccumulation and inflammatory responses in fish tissues (Zhao et al., 2020, 2022). Arsenic (As) exists widely in nature in both organic and inorganic forms. Arsenic trioxide (As_2_O_3_), an inorganic As, is highly toxic and can damage living organisms (Guo et al., 2019). In recent years, some countries have reported high As concentrations in water resources, which pose a serious threat to aquatic organisms and humans. A study of As-polluted water bodies found that the average concentration of As in the lower Songhua River in China was 9.716 ± 4.977 μg/g (Liu et al., 2018), and a survey reported that the groundwater As concentration in North Dakota exceeded the US environmental protection safety standard (10 μg/L) (Powers et al., 2019). Groundwater is also a water resource for fish farming, which may lead to a human exposure to As through water and diet. For example, in some polluted areas of Norway, the As level in fish is as high as 110 μg/g (Julshamn et al., 2012). In Guizhou, China, arsenic pollution from coal fire causes damage to human skin (Zeng et al., 2021). An investigation found that drinking water contaminated with high concentrations of As causes human As poisoning and pathological damage to skin (Jin et al., 2003). At a non-lethal As exposure level (2.83 mg/L) *in vivo*, As induces severe pathological lesions and disturbs the renal function in common carp (Wang et al., 2020). A study proved that the toxic heavy metal lead induces an intestinal damage in common carp (Zhang et al., 2021). Common carp is an important edible economic fish in China and is widely farmed for its tasty meat and high nutritional value. However, the mechanism underlying the As-induced damage to the carp intestine remains unclear.

Recent findings demonstrate that excess levels of the heavy metal manganese increase the *Bacteroidetes* abundance, decrease the *Firmicutes/Bacteroidetes* ratio, and increase the lipopolysaccharide (LPS) levels (Tinkov et al., 2021). LPS secreted by gram-negative bacteria can lead to tissue damage (Cheng et al., 2018). Studies have also shown that lead exposure induces intestinal inflammation and increases the LPS level in serum (Zhang et al., 2021). LPS treatment induces inflammatory reactions and causes a barrier breakdown in alveolar epithelial cells (AECs) (Shi et al., 2022). Recent studies have shown that gram-negative bacterial treatment causes intestinal epithelial dysfunction, increases the permeability of intestinal epithelial cells, and increases the LPS level in serum (Stephens and Weid, 2022). However, whether As can cause changes in the intestinal microflora of common carp and destroy the structural integrity of intestines in fish remains unclear.

Probiotics are living microorganisms that are beneficial to health (Hu et al., 2020). Recent studies have shown that the intestinal microbiota plays a key role in maintaining animal and human health (Ye et al., 2021). Recent findings have indicated that environmental pollutants, such as antibiotics, heavy metals, and industrial wastewater, can affect the composition of the intestinal flora, lead to physiological dysfunction, and even cause certain diseases (Jin et al., 2017; Miao et al., 2022). Exposure to mercury increases the levels of *Aeromonas hydrophila* and *Aeromonas sobria* in the carp intestine and thus affects the intestinal health and increases the levels of pathogenic bacteria (Shang et al., 2022). Therefore, it is particularly important to maintain a healthy intestinal flora.

The intestinal mucosal barrier plays an important role in preventing hazardous substances from penetrating the intestine and entering the blood (Lopetuso et al., 2015). Tight junctions are the most important components of the intestinal mucosal barrier (Zhang et al., 2021; Zhou et al., 2023) and play an important role in its function. The proteins of the zona occludens (ZO) family are among the most important tight junction proteins (Lopetuso et al., 2015). ZO-1 is closely related to intestinal mucosal barrier function and participates in the passage of ions and small soluble molecules (Zhang et al., 2021). Damage to ZO-1 obviously affects the formation of tight junctions and the normal function of the defense barrier (Cario et al., 2004). Occludin is an integral membrane protein that is closely associated with tight junctions in epithelial and endothelial cells and has been implicated in the damage to tight junctions (Zhang et al., 2021). However, the effect of As on the common carp intestinal microbe composition and the effect of As on the intestinal structure through the tight junction protein *ZO-1* and *occludin* genes have rarely been investigated. This study aimed to establish a common carp model of As poisoning, to explore the negative effects of As on the intestinal structural integrity and intestinal microbiota diversity of common carp, and to provide new insights into the toxic mechanism through which As damages the intestinal barrier.

## 2. Materials and methods

### 2.1. Animals

In total, 90 healthy common carp juveniles (*Cyprinus carpio*, weighing 45–50 g) were obtained from the Hulan Experimental Station of Heilongjiang Fisheries Research Institute (Harbin, China), Chinese Academy of Fishery Sciences, and were transferred to the laboratory. Before exposure to sodium arsenite (NaAsO_2_), the fish were acclimated to 23 ± 1^°^C with a light cycle of 14 h light/10 h dark for 1 week. The fish were given commercial feed (Tongwei Co., Ltd., Chengdu, China) twice daily. The date of manufacture of the feed was 22 July 2022, and its shelf life was 3 months. The composition of the feed contained 35% crude protein, 4.0% crude fat, and 15% crude ash. The animal experiment was performed according to the Guidelines for the Feeding and Application of Laboratory Animals of Heilongjiang Fisheries Research Institute, Chinese Academy of Fishery Sciences, and was approved by the Committee on the Ethics of Animal Experiments of Heilongjiang Fisheries Research Institute, Chinese Academy of Fishery Sciences (HLR-03).

### 2.2. Test chemicals

Sodium arsenite (NaAsO_2_) was purchased from Sigma-Aldrich (St. Louis, MO, USA). An LPS ELISA kit was obtained from ELISA Bio-Co., Ltd., Jiangsu, China. The TRIzol reagent was purchased from Life Technologies (CA, USA). A BCA assay kit was purchased from Solarbio (Beijing, China). A SYBR-Green Real-Time PCR kit and cDNA reverse transcription kit were obtained from Takara (Dalian, China). Primary antibodies (occludin and ZO-1) were purchased from Jiangsu Parent Biology Research Center Co., Ltd. (China), and β-actin was obtained from Proteintech (China).

### 2.3. Experimental design

In total, 90 healthy common carps were randomly divided into two groups (the control group and the As group), with three tanks for each group and 15 fish per tank. The carp were maintained and fed in 180-L glass tanks at 23 ± 1^°^C for 30 days. Half of the water was replaced with dechlorinated drinking water every day. The carp in the As group received 2.83 mg/L As, and the arsenic dose was determined based on previous studies (Liu et al., 2018; Wang et al., 2021). The 96 h LC50 value of As_2_O_3_ for Indian Major Carp is 28.30 mg/L (Vutukuru et al., 2007), and 10% of the 96 h LC50 is a commonly used test concentration in toxicology studies. Ten milliliters of stock solution with an As concentration of 25.47 mg/mL was prepared, and 100 μl of stock solution was added after each time that half of the water was replaced. The actual As concentration in the As group was 2.826 ± 0.032 mg/L. The physical and chemical parameters of the water in the tank were monitored daily (temperature: 23 ± 1^°^C, dissolved oxygen: 6.50 ± 0.22 mg/L; pH: 7.3 ± 0.3; and ammonia: 0.16 ± 0.03 mg/L). Oxygen supplementation using an air stone was performed during the experimental period to ensure enough dissolved oxygen in the water.

On the 30th day of the experiment, six fish were randomly selected from each tank of the control and As groups. A total of 36 fish were euthanized with MS-222 (100 mg/L, Sigma, USA). Blood was collected by caudal venipuncture, and all the fish were then euthanized. Part of the collected blood was used to measure the accumulation of As in blood; the other part of the collected blood was placed in a 4^°^C refrigerator for 24 h, and the supernatant (serum) was obtained. The livers, muscles, distal intestinal tissues, and intestinal contents were collected. The livers, muscles, and part of the distal intestinal tissues were immediately frozen in liquid nitrogen and then transferred to a −80^°^C freezer for subsequent experiments. Part of the distal intestinal tissues were fixed with 10% formaldehyde for a histological study.

### 2.4. Histological study

The intestinal tissues from the control and As-treated fish were fixed with 10% formaldehyde for a routine histological observation. The fixed intestinal tissues were washed with 70% ethanol several times, dehydrated via a graded series of ethanol, and embedded in paraffin (Sun et al., 2022). Paraffin-embedded tissues were serially sectioned at a thickness of 5 μm and stained with hematoxylin and eosin. The stained intestinal sections were observed under an Axioskop microscope (IX71, Olympus), and the intestinal sections were quantitatively analyzed using the software program provided with the Axioskop microscope system.

### 2.5. Assays of the As content in tissues and LPS content in serum

The liver, muscle, intestinal, and blood samples (0.1 g) were placed in polytetrafluoroethylene (PTFE) microwave digestion tanks, and 5 mL of HNO_3_ (concentration of 65%) solution and 1 mL of H_2_O_2_ (concentration of 30%) solution were sequentially added. The digestion tanks were sealed and placed in a microwave digester, and the samples were mineralized at 185^°^C for 14.5 min. The As levels in the carp livers, intestines, muscles, and blood were determined by inductively coupled plasma-mass spectrometry (ICP-MS; Thermo iCAPQ, USA). The LPS levels in the carp serum were measured with an LPS kit according to the manufacturer’s instructions. Briefly, the prepared samples were placed in a 96-well plate, and the optical density of the samples in the 96-well plate at 545 nm was analyzed using a microplate reader (BioTek, Winooski, VT, USA) according to the manual.

### 2.6. Quantitative real-time polymerase chain reaction

The intestinal tissues were removed from the −80^°^C refrigerator, immediately placed in a cooled mortar, and ground while liquid nitrogen was added to the mortar. Total RNA from three separate carp intestinal tissues from each group was isolated with TRIzol reagent according to the manufacturer’s protocol. The quality of total RNA was checked using a NanoDrop 2000 Spectrophotometer based on an A260/280 ratio in the range of 1.70–1.90. cDNA was synthesized according to the manufacturer’s instructions (Tiangen, Beijing, China), and β-actin was used as an internal reference gene. The specific primer sequences used for qRT-PCR are listed in Table 1. Primer information was obtained from a previous study (Zhang et al., 2021; Yu et al., 2022). qRT-PCR was performed using SYBR Green and a LightCycler^®^ 96 instrument (Roche, Switzerland). The reaction programme was as follows: denaturation at 95^°^C for 5 min and 40 cycles of 95^°^C for 10 s, 59^°^C for 10 s, and 72^°^C for 30 s. The fluorescence signal was measured at the annealing/extension step. The relative abundance of mRNA was calculated using the 2^–ΔΔ*Ct*^ method.

**TABLE 1:** Primers used in this study.

β-actin	Forward reverse	GCTATGTGGCTCTTGACTTCG CCGTCAGGCAGCTGATAGCT
ZO-1	Forward reverse	CCGAAGCTTTGACAGCAAAC GGTTGATCTTCTCCACTGACTC
Occludin	Forward everse	GACGCCATGGATGAGTACAA GTGGTTGAGTTTGGCTTTCAG

### 2.7. Western blot analysis

First, a BCA assay kit was used to determine the protein concentration (50 mg of tissues, *n* = 3/group). Proteins were separated by 15% SDS-polyacrylamide gel electrophoresis (PAGE) and transferred onto polyvinylidene fluoride membranes. The membranes were then incubated with the corresponding primary antibodies at 1:1000 dilution with TBST at 4^°^C overnight and then with goat anti Rabbit IgG secondary antibody (1:1000, A0208, Beyotime, China) at 37^°^C for 1.5 h. β-Actin served as a loading control for relative protein expression. Hypersensitive ECL Chemiluminescent Liquid (P0018AS, Beyotime, China) was then added to the membranes. Protein band images were obtained using a Clinx ChemiScope fluorescence and chemiluminescence imaging system. The protein band optical density (OD) value was calculated using ImageJ software (Xu et al., 2022). The relative protein level of each detected protein was calculated according to the following formula: the OD value of the detected protein/the OD value of β-actin (reference protein) (Cui et al., 2022).

### 2.8. DNA extraction and 16S rRNA gene analysis

After 30 days of exposure to As, six samples of the carp intestinal contents were randomly collected from different water tanks (three samples from the control group and three samples from the As group) (Song et al., 2022), immediately frozen in liquid nitrogen, and stored in a −80^°^C freezer. Microbial DNA was extracted from the carp intestinal contents with the QIAamp DNA Stool Mini Kit (Qiagen, Germany). PCR amplification was performed with universal primers for the 16S variable region V3–V4. The V3–V4 variable region primer pairs F (5′-CCTACGGRRBGCASCAGKVRVGAAT-3′) and R (5′-GGACTACNVGGGTWTCTAATCC-3′) were used for amplification of the gut microbiota 16S rRNA gene (Logue et al., 2016). Raw reads were analyzed using the Quantitative Insights Into Microbial Ecology (QIIME) toolkit (version 1.17). An operational taxonomic unit (OTU) is a unified notation that is artificially set by phylogeny to facilitate analysis. In this analysis, the OTUs were clustered with a 97% similarity cutoff using UPARSE, and chimeric sequences were identified and removed using UCHIME. The classification of each 16S rRNA gene sequence was analyzed using the RDP classifier against the SILVA (SSU115) 16S rRNA database based on a 70% confidence threshold.

### 2.9. Statistical analysis

Statistical analysis was performed using SPSS 20.0 software. All the data are presented as the means ± SDs and were tested by one-way ANOVA. Statistical significance was assumed if *P* < 0.05.

## 3. Results

### 3.1. Effect of as exposure on intestinal histopathology

**FIGURE 1:**
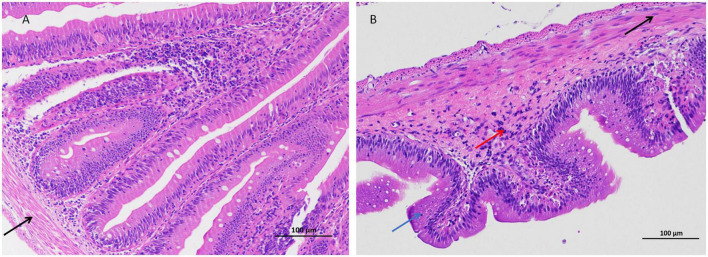
Arsenic caused distal intestinal histopathological changes (200 × magnification). **(A)** Control group, **(B)** arsenic (As)-treated group. Blue arrows (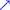): shortened intestinal villi, right black arrow (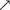): thinner muscular layer, and red arrows (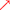): inflammatory cell infiltration.

In this study, the intestinal histopathological changes in fish belonging to different experimental groups after feeding for 30 days were obtained, as shown in Figure 1. The results showed that As exposure caused histopathological changes in intestinal tissues, and these effects included shortened intestinal villi, a thinner muscular layer of the intestine (Figure 1 and Table 2), and infiltration of inflammatory cells (Figure 1).

**TABLE 2:** Changes in the intestinal villi and muscular layer in distal intestinal tissues.

	Control group	Arsenic-treated group
Villi length (μm)	432.75 ± 3.68	218.64 ± 7.62*
Muscular layer thickness (μm)	35.28 ± 2.64	21.18 ± 2.73*

*P < 0.05.

### 3.2. Accumulation of As in tissues and LPS level in serum

**FIGURE 2:**
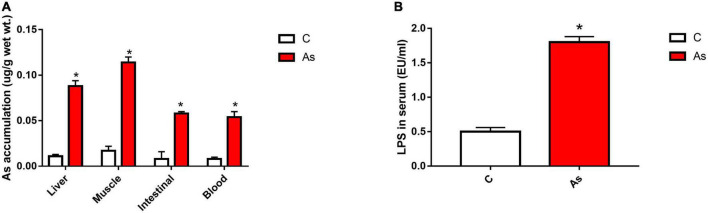
Effect of arsenic (As) exposure on As accumulation in tissues **(A)** and lipopolysaccharide (LPS) levels in serum **(B)**. The data are presented as the means ± standard deviations of three parallel measurements. *P < 0.05 indicates a significant difference between the two groups.

The accumulation of As in the liver, intestines, muscles, and blood was detected (Figure 2A). The data showed that the accumulation of As in all of the above-mentioned tissues was significantly (*P* < 0.05) higher in the As-treated group than in the control group. The level of As accumulation in the above-mentioned carp tissues was placed in the order of muscles > livers > intestines > blood.

After 30 days of exposure to As in water, the LPS level in the carp serum was measured, and the LPS level in the As group was found to be significantly higher (*P* < 0.05) than that in the control group (Figure 2B).

### 3.3. Effect of As exposure on tight junction-related genes and proteins

**FIGURE 3:**
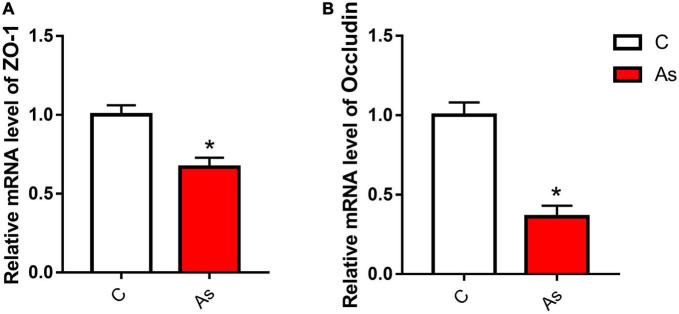
Effect of arsenic (As) exposure on zonula occludens (ZO-1) **(A)** and occludin **(B)** expression. The data are presented as the means ± standard deviations of three parallel measurements. *P < 0.05 indicates a significant difference between the two groups.

**FIGURE 4:**
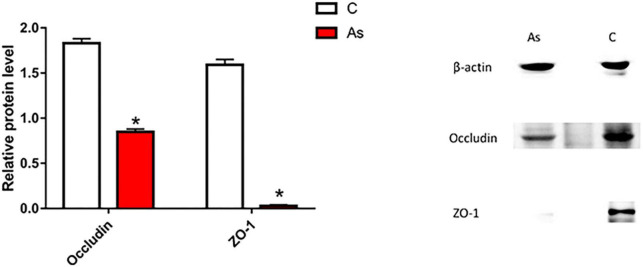
Effect of arsenic (As) exposure on zonula occludens (ZO-1) and occludin protein levels. The data are presented as the means ± standard deviations of three parallel measurements. *P < 0.05 indicates a significant difference between the two groups.

First, the expression of tight junction-related genes in the carp intestine was measured by qRT-PCR. The melting curve for each PCR product showed only one peak. The results are presented in Figure 3 and show that the levels of the tight junction-related genes *ZO-1* and *occludin* were significantly (*P* < 0.05) decreased after exposure to As. The expression of tight junction proteins was then detected by Western blotting, and the results are given in Figure 4. Arsenic exposure inhibited the expression of the tight junction proteins ZO-1 and occludin in carp intestines.

### 3.4. Sequencing data

**FIGURE 5:**
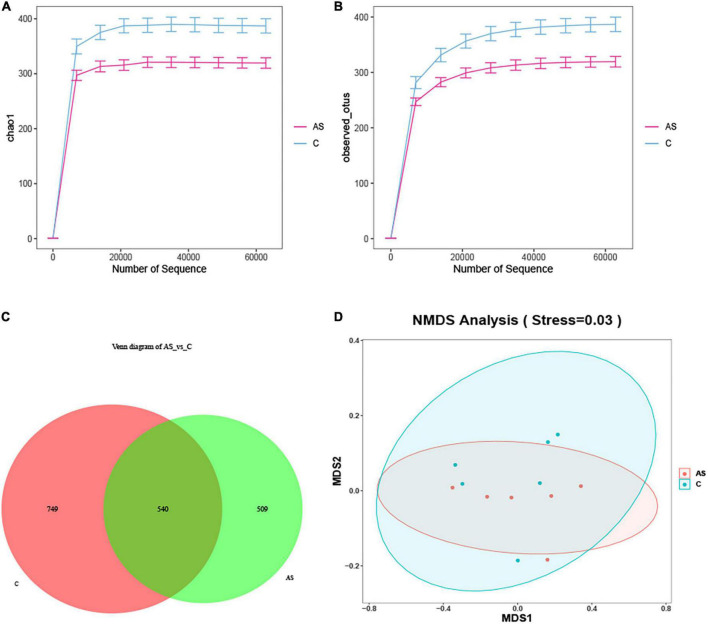
Changes in the intestinal microbiota in carp after arsenic (As) exposure. **(A)** Chao sparsity curve. **(B)** Sparse curves of operational taxonomic units (OTUs). **(C)** Venn diagram. **(D)** Multidimensional scaling (NMDS) of fecal microbiota differences between the healthy fish and the As-treated fish.

The microbial diversity was investigated by 16S rRNA. As shown in Figure 5, the rarefaction curve obtained demonstrated that the sequencing data were reliable (Figures 5A, B). The amplicon sequence variant (ASV) ID indicated the ID of the ASV feature sequence after QIIME2 denoising. An ASV sample sheet with a similar level of 100% was selected for analysis. The number of ASVs per sample was obtained, out of which 1,289 and 1,049 ASVs were found for the control and As groups, respectively. The two groups shared 540 ASVs. In addition, 749 ASVs in the control group did not belong to the As-treated group, and 509 ASVs in the As-treated group did not belong to the control group (Figure 5C). An NMDS analysis was performed, and the results showed that the flora in the control and As groups belonged to different categories (Figure 5D), demonstrating differences in the microbial diversity between the control group and the treatment group.

### 3.5. Comparisons at the phylum and genus levels

**FIGURE 6:**
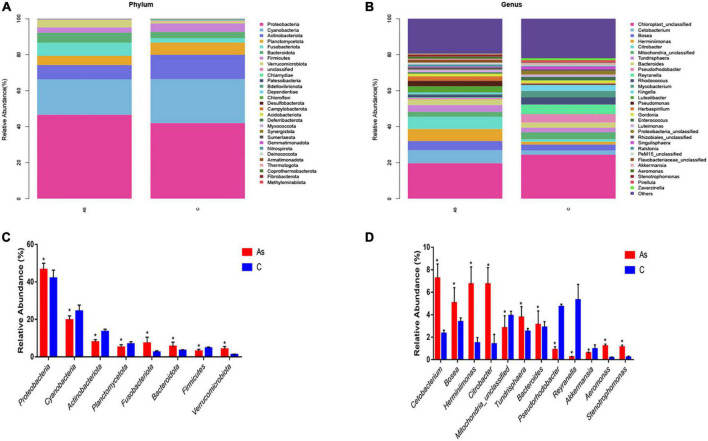
Relative abundance and differences in the intestinal flora at the phylum and genus levels. **(A,B)** Relative abundance and differences at the phylum level, respectively. **(C,D)** Relative abundance and differences at the genus level, respectively. *P < 0.05 indicates a significant difference between the two groups.

All sequences were identified at the phylum level and at the genus level. At the phylum level, the data showed that *Proteobacteria*, *Cyanobacteria*, *Actinobacteriota*, *Planctomycetota*, *Bacteroidota*, and *Firmicutes* were the six most dominant flora in the control group. *Proteobacteria*, *Cyanobacteria*, *Actinobacteriota*, *Planctomycetota*, *Fusobacteriota*, and *Bacteroidota* were the six most dominant flora in the As group. Arsenic treatment significantly (*P* < 0.05) increased the levels of the intestinal microbes *Proteobacteria*, *Fusobacteriota*, *Bacteroidota*, and *Verrucomicrobiota* in contrast and significantly (*P* < 0.05) decreased those of *Cyanobacteria*, *Actinobacteriota*, *Planctomycetota*, and *Firmicutes* (Figures 6A, B).

At the genus level, the six most abundant intestinal microbiota in the control group were *Chloroplast_unclassified, Pseudorhodobacter*, *Reyranella*, *Mitochondria_unclassified*, *Rhodococcus*, and *Mycobacterium*. *Chloroplast_unclassified*, *Cetobacterium*, *Bosea*, *Tundrisphaera*, *Herminiimonas*, and *Citrobacter* were the six most predominant intestinal microbiota in the As group. Significantly (*P* < 0.05) higher levels of *Citrobacter*, *Pseudorhodobacter*, *Bacteroides*, *Aeromonas*, and *Stenotrophomonas* and significantly (*P* < 0.05) lower levels of *Reyranella*, *Akkermansia*, and *Pseudorhodobacter* were found in the As group compared with the control group (Figures 6C, D).

### 3.6. Significantly different species identified by LEfSe

**FIGURE 7:**
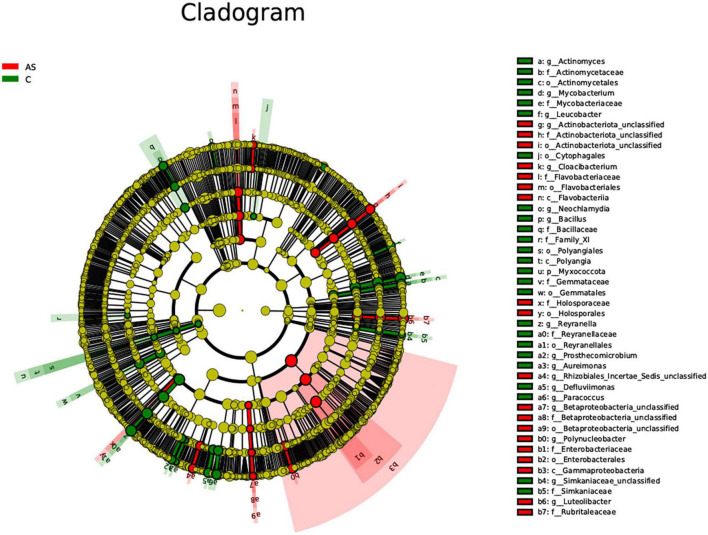
All characteristic species were detected by LDA Effect Size (LEfSe) analysis using the Kruskal-Wallis rank sum test (calculated by Wilcoxon signed-rank test, Linear discriminant analysis (LDA) score >3, and *P* < 0.05 significance).

The LEfSe method was used to explore significantly different species. At the phylum level, the *Bacteroidota* level was significantly (*P* < 0.05) increased in the As group compared with the control group. At the family level, the As group showed significantly (*P* < 0.05) higher levels of *Flavobacteriaceae*, *Sphingobacteriaceae*, *Betaproteobacteria*, and *Betaproteobacteria_unclassified* and significantly (*P* < 0.05) lower levels of *Mycobacteriaceae*, *Actinomycetaceae*, and *Bacillaceae* compared with the control group (Figure 7).

## 4. Discussion

As-containing pesticides, As-containing herbicides, and defoliants are widely used in agricultural production and can affect aquatic ecosystems. The As pollution of aquatic ecosystems is a serious environmental problem. Studies have shown that when As is added to the diet of mice, it can accumulate in the liver and intestines (Jankong et al., 2007; Coryell et al., 2018; Li et al., 2021). Arsenic accumulation has been found in the intestine and muscles of *Oreochromis mossambicus* after dietary exposure to As (Song et al., 2022). The results of a geographical survey showed that the distribution of As in *Salmo trutta* tissues follows the order of kidney > liver > gill > fin > gonad > muscle. In our experiment, we found As accumulation in muscles, livers, intestines, and blood, and the order of the As concentration was muscle > liver > intestine > blood. Arsenic enters the body of a fish in two ways: through the consumption of As in water and through a direct contact with As in water. After exposure to As in water, the eyes and skin are in direct contact with As in water, which makes the eyes and skin directly absorb As from the water, leading to a high As accumulation in the eyes and skin of zebrafish (Hallauer et al., 2016). Common carp have no scales, and As in water can enter muscles through the skin. Zebrafish have scales, and these scales can block the absorption of As by skin. Therefore, As accumulation in zebrafish muscles is lower than that in other tissues, whereas As accumulation in common carp muscles is found to be higher than that in other tissues, but this finding needs to be further explored.

The intestine can digest, absorb, and excrete As. Arsenic in intestinal contents can affect the composition and diversity of intestinal microbes. Therefore, a carp model of As-induced poisoning was established in this study, and an Illumina high-throughput sequencing technology was used to detect the structure and diversity of the intestinal microbes of common carp. The results of our study indicated that the exposure to As affected the intestinal flora. Adverse changes in the intestinal flora can impair normal physiological functions of the body and induce various diseases (Li et al., 2022; Nicholson et al., 2012). In addition, intestinal damage was observed in our study, and As accumulation in the intestine was found under As exposure, which indicates that As damaged the intestines and impaired the intestinal barrier by affecting the intestinal flora in common carp. This study revealed that 30 days of exposure to As decreased the intestinal bacterial diversity of common carp. A growing body of evidence suggests that the development of many diseases, such as inflammatory bowel disease, is associated with a decrease in the intestinal microbiota diversity (Zhu et al., 2018; Zhou et al., 2019). In this study, the data showed that *Proteobacteria*, *Cyanobacteria*, *Actinobacteriota*, *Planctomycetota*, *Bacteroidota*, and *Firmicutes* were the six most important intestinal flora of common carp. *Bacteroidota* is a gram-negative bacterium that produces LPS, which can induce an inflammatory response in the body, and increased LPS levels can cause an impairment of the intestinal barrier function (Youmans et al., 2015). *Aeromonas* are also zoonotic pathogens that can opportunistically infect fish and are responsible for significant losses in fish aquaculture worldwide (Ljubobratovic et al., 2017). Supplementation with the probiotic *Lactobacilli* reduces the number of harmful bacteria, such as *Aeromonas* (Ljubobratovic et al., 2017). In this study, a significantly higher abundance of *Bacteroidota* at the phylum level was found in the intestine of As-treated carp compared with that of the control carp. Moreover, at the genus level, higher abundances of *Aeromonas* and *Stenotrophomonas* were found after As treatment in our experiment. Our above-described findings indicated that excess As increased the presence of harmful bacteria and the production of LPS and impaired the intestinal barrier in common carp. A study conducted by Youmans et al. (2015) also supports our findings and had demonstrated that the decrease in the ratio of *Firmicutes* to *Bacteroidetes* is related to the development of diarrhea (Youmans et al., 2015). *Actinobacteriota* is a gram-positive bacterium that plays a key role in the maintenance of a normal intestinal function and is used in aquaculture to prevent certain diseases (Alvarez et al., 2017). *Akkermansia* is a very important probiotic endowed with the ability to treat many diseases (Dao et al., 2016). This study found that the exposure to As reduced the abundance of the probiotic *Actinobacteriota* at the phylum level and reduced the abundance of the probiotic *Akkermansia* at the genus level, indicating that As reduces the protective function of beneficial microbes on intestines.

Zonula occludens-1 (ZO-1) and occludin are the most important tight junction factors that play critical roles in maintaining the intestinal epithelial barrier (Bazzoni et al., 2000). Decreased gene expression of the tight junction proteins, such as ZO-1 and occludin, indicates that the intestinal physical barrier function is weakened (Takiishi et al., 2017). In this study, As exposure reduced the expression of ZO-1 and occludin at both the protein and gene levels, which indicated that As caused an intestinal barrier damage in common carp by decreasing the levels of ZO-1 and occludin. The results from previous studies are consistent with our findings and showed that LPS can reduce the expression of tight junction proteins, such as *ZO-1* and *occludin*, leading to an impaired intestinal barrier (Ruan et al., 2014; Zhang et al., 2021; Du et al., 2022). *Actinobacteriota* can promote the expression of tight junction-related factors (Purchiaroni et al., 2013). Additionally, in our study, the LPS levels in serum increased after As treatment, further demonstrating that As exposure impaired the intestinal barrier and that LPS entered the blood in common carp. In conclusion, As treatment impaired the intestinal barrier in common carp, increased the abundance of harmful bacteria, decreased the abundance of beneficial bacteria, and reduced the intestinal microbiota diversity. Moreover, decreased gene and protein expression levels of the tight junction-related factors ZO-1 and occludin were detected after the development of an intestinal injury caused by As. Excess As increased the LPS levels in serum, and exposure to As resulted in As accumulation in the muscles, livers, intestines, and blood. The present study also provided the first demonstration that the As concentration in organs followed the order muscle > liver > intestine > blood, although this finding needs to be investigated in the future. Taken together, the results of this study provide a basis for serving as a link between microbial changes in the intestine and As toxicity. When the concentration of As in the water environment becomes too high, the common carp can mitigate the damage caused by As to the intestine through its diet, and the diet should have the ability to improve the intestinal microbial diversity and reduce the serum LPS concentrations.

## Data availability statement

The datasets presented in this study can be found in online repositories. The names of the repository/repositories and accession number(s) can be found below: NCBI–PRJNA941446.

## Ethics statement

This animal study was reviewed and approved by Guidelines for the Feeding and Application of Laboratory Animals of Heilongjiang Fisheries Research Institute, Chinese Academy of Fishery Sciences, and was approved by the Committee on the Ethics of Animal Experiments of Heilongjiang Fisheries Research Institute, Chinese Academy of Fishery Sciences (HLR-03). Written informed consent was obtained from the owners for the participation of their animals in this study.

## Author contributions

XShi: writing—review and editing, conceptualization, software, and data curation. WX: writing—review and editing, methodology, and data curation. XC: data curation and investigation. JC: methodology and data curation. XSha: writing—review and editing, conceptualization, software, and data curation. XT: investigation. ZJ: supervision and writing-review and editing.
