# Supplementary figures and images for: Effect of arsenic stress on the intestinal structural integrity and intestinal flora abundance of Cyprinus carpio
Source: Front Microbiol. 2023 Apr 24;14:1179397. doi: 10.3389/fmicb.2023.1179397 (PMC10165157; doi:10.3389/fmicb.2023.1179397)

# Phylum

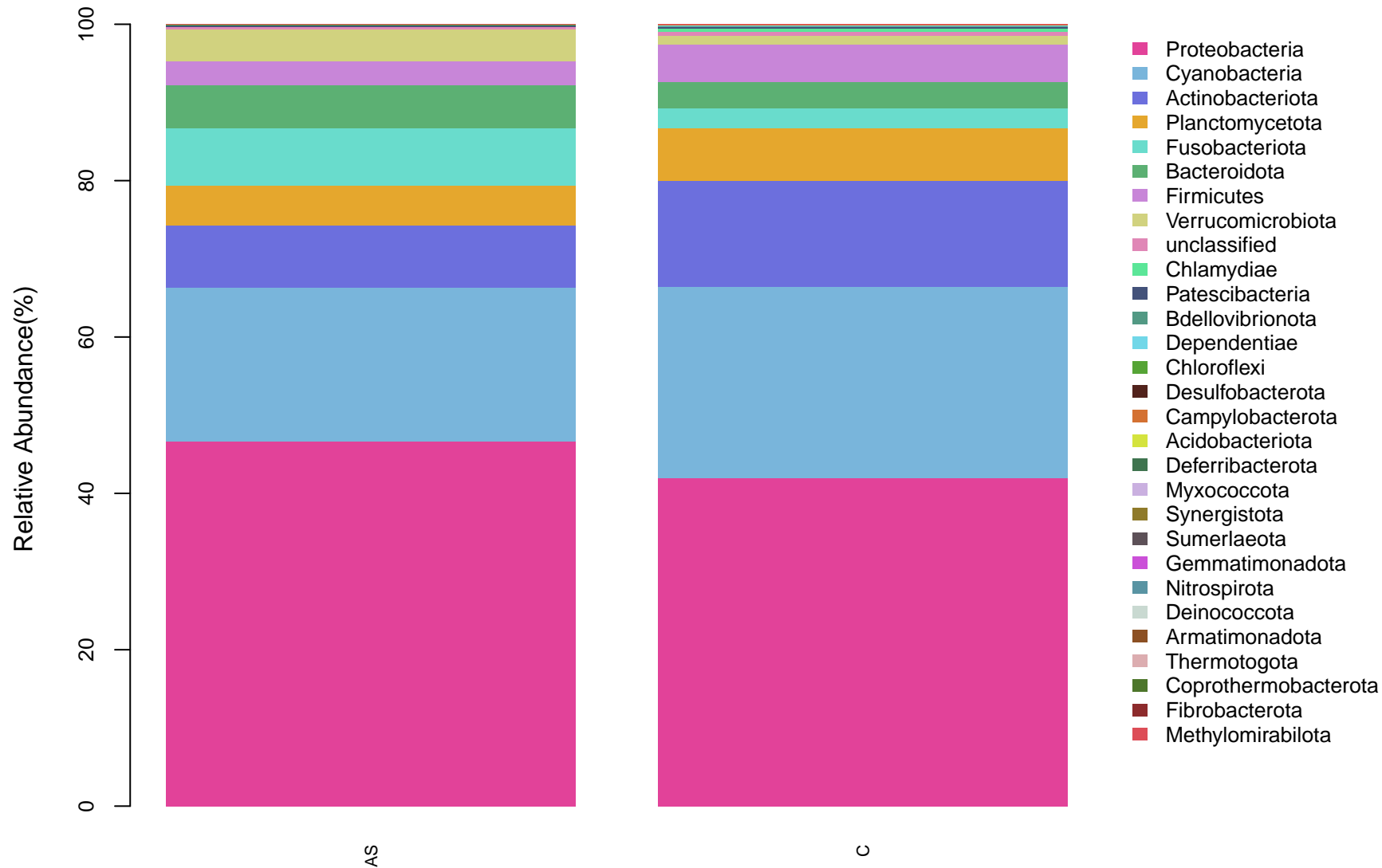

Supplement: Supplementary file 1 [file Data_Sheet_1.PDF]

# Genus

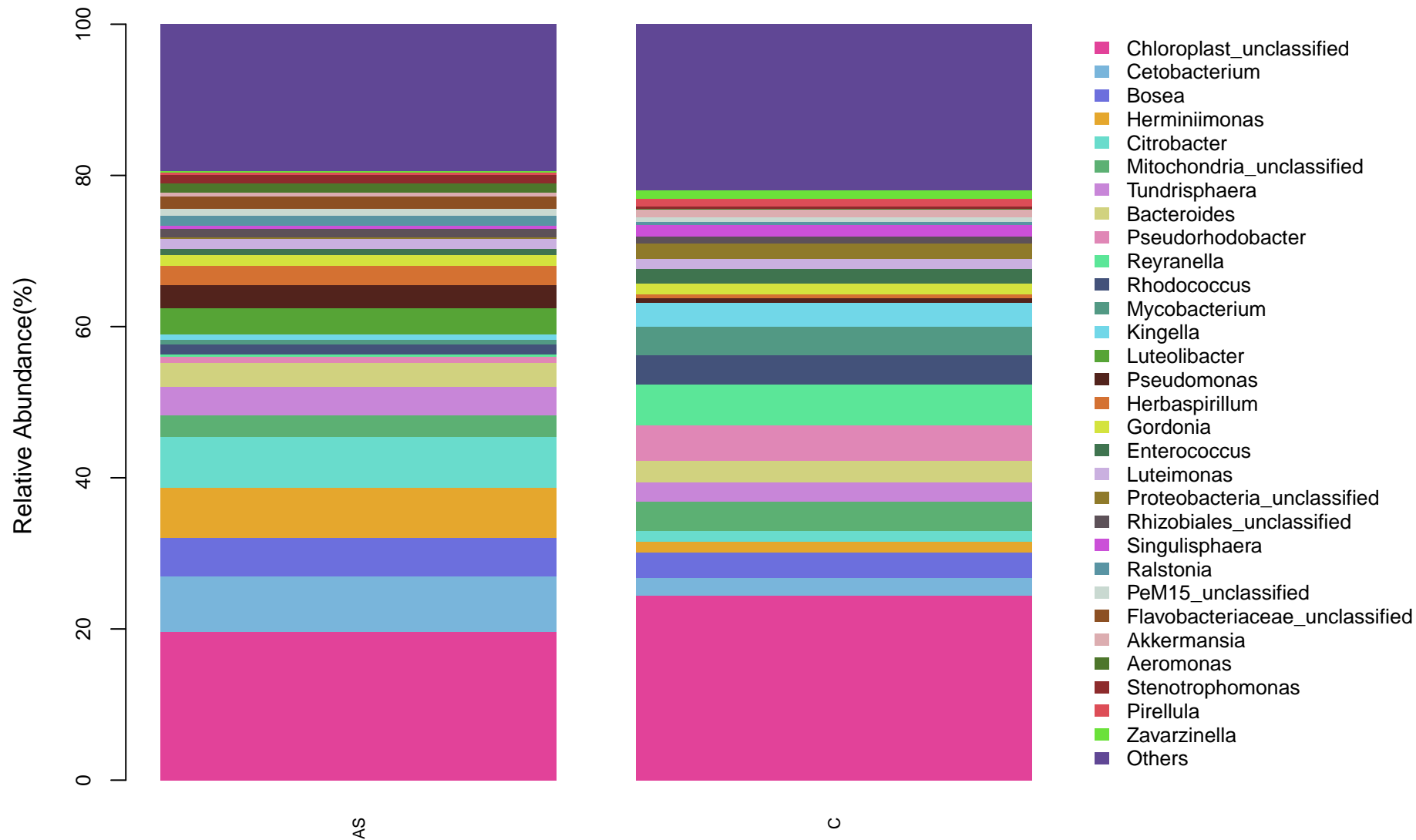

Supplement: Supplementary file 2 [file Data_Sheet_2.PDF]
