# Supplementary material for: Effect of arsenic stress on the intestinal structural integrity and intestinal flora abundance of Cyprinus carpio
Source: Front Microbiol. 2023 Apr 24;14:1179397. doi: 10.3389/fmicb.2023.1179397 (PMC10165157; doi:10.3389/fmicb.2023.1179397)
Supplement: Supplementary file 3 [file Data_Sheet_3.PDF]

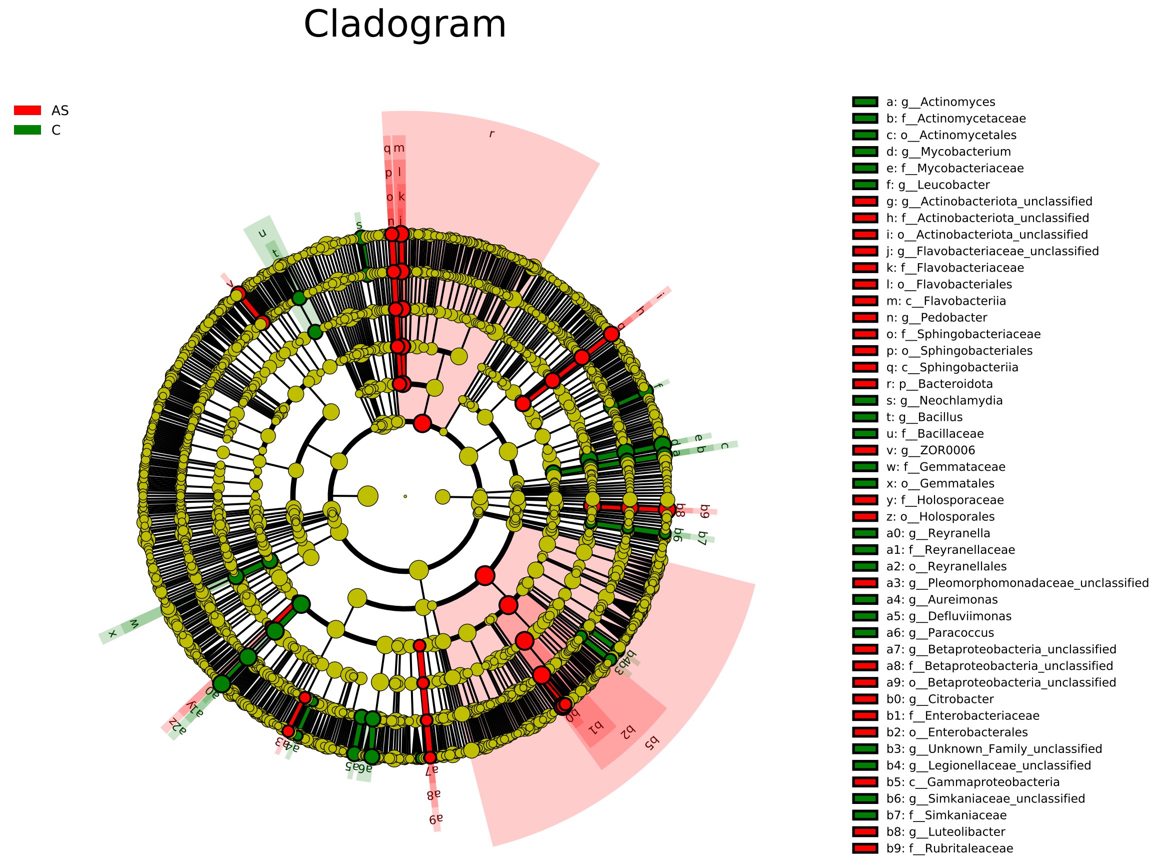

Cladogram
AS
C
a: g__Actinomyces
b: f__Actinomycetaceae
c: o__Actinomycetales
d: g__Mycobacterium
e: f__Mycobacteriaceae
f: g__Leucobacter
g: g__Actinobacteriota_unclassified
h: f__Actinobacteriota_unclassified
i: o__Actinobacteriota_unclassified
j: g__Flavobacteriaceae_unclassified
k: f__Flavobacteriaceae
l: o__Flavobacteriales
m: c__Flavobacteriia
n: g__Pedobacter
o: f__Sphingobacteriaceae
p: o__Sphingobacteriales
q: c__Sphingobacteriia
r: p__Bacteroidota
s: g__Neochlamydia
t: g__Bacillus
u: f__Bacillaceae
v: g__ZOR0006
w: f__Gemmataceae
x: o__Gemmatales
y: f__Holosporaceae
z: o__Holosporales
a0: g__Reyranella
a1: f__Reyranellaceae
a2: o__Reyranellales
a3: g__Pleomorphomonadaceae_unclassified
a4: g__Aureimonas
a5: g__Defluviimonas
a6: g__Paracoccus
a7: g__Betaproteobacteria_unclassified
a8: f__Betaproteobacteria_unclassified
a9: o__Betaproteobacteria_unclassified
b0: g__Citrobacter
b1: f__Enterobacteriaceae
b2: o__Enterobacterales
b3: g__Unknown_Family_unclassified
b4: g__Legionellaceae_unclassified
b5: c__Gammaproteobacteria
b6: g__Simkaniaceae_unclassified
b7: f__Simkaniaceae
b8: g__Luteolibacter
b9: f__Rubritaleaceae
