# Supplementary material for: Effect of arsenic stress on the intestinal structural integrity and intestinal flora abundance of Cyprinus carpio
Source: Front Microbiol. 2023 Apr 24;14:1179397. doi: 10.3389/fmicb.2023.1179397 (PMC10165157; doi:10.3389/fmicb.2023.1179397)

Arsenic stress

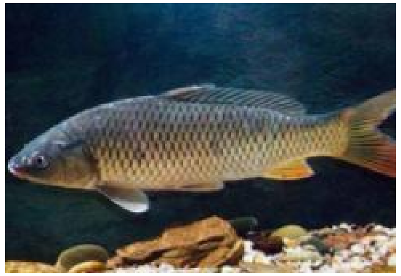

Intestinal tissue structure and Serum

16S rRNA

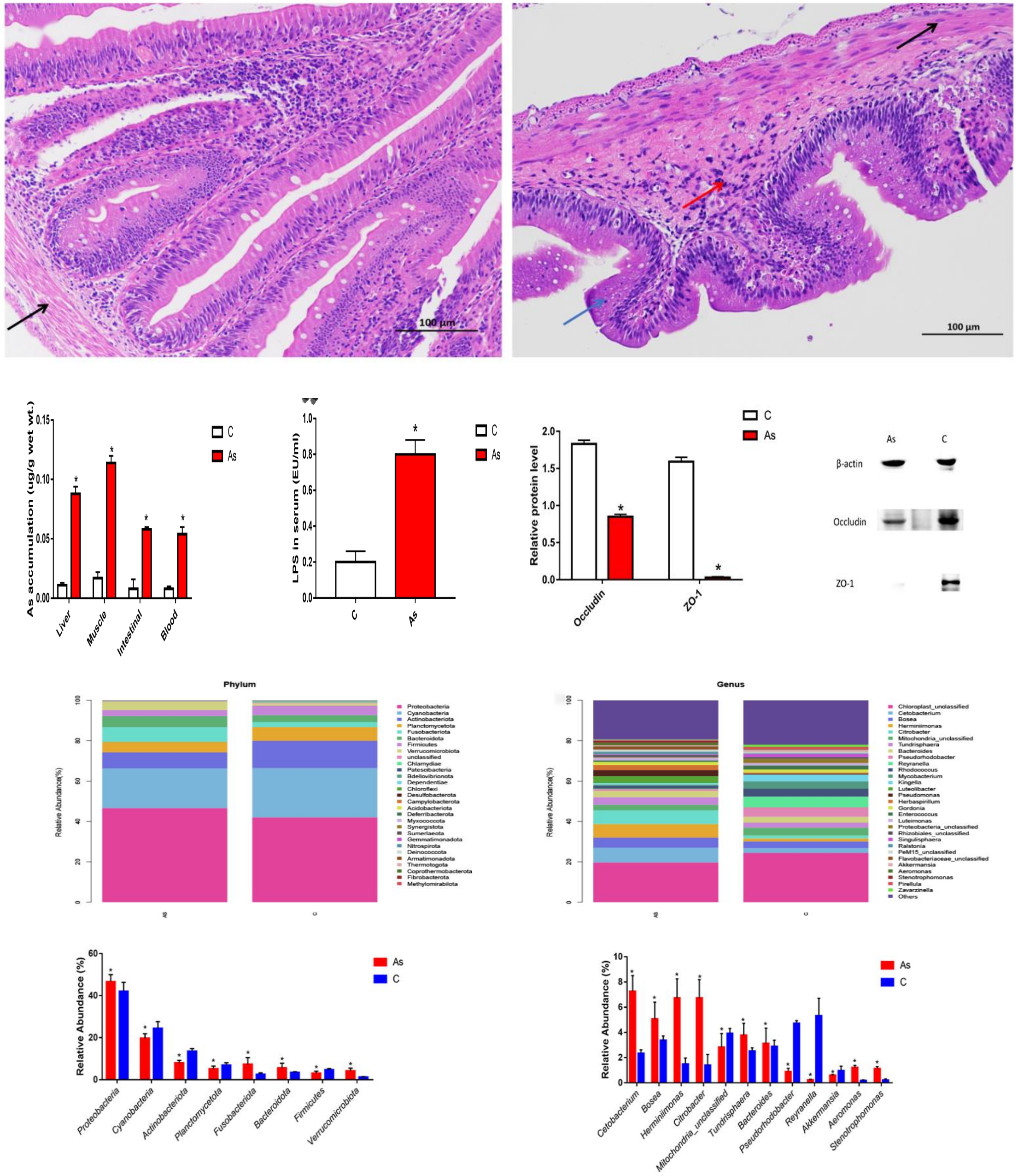

Supplement: Supplementary file 4 [file Data_Sheet_4.PDF]
